# Mobile phone thermal imaging as an ambulatory assessment tool in Raynaud’s phenomenon

**DOI:** 10.1093/rheumatology/kead210

**Published:** 2023-05-15

**Authors:** Graham Dinsdale, Shanees Nazeer, Joanne Manning, Andrea Murray, Ariane Loraine Herrick

**Affiliations:** Department of Rheumatology, Salford Royal Hospital, Northern Care Alliance NHS Foundation Trust, Salford, UK; Centre for Musculoskeletal Research, School of Biological Sciences, The University of Manchester, Manchester Academic Health Science Centre, Manchester, UK; Centre for Musculoskeletal Research, School of Biological Sciences, The University of Manchester, Manchester Academic Health Science Centre, Manchester, UK; Department of Rheumatology, Salford Royal Hospital, Northern Care Alliance NHS Foundation Trust, Salford, UK; Centre for Musculoskeletal Research, School of Biological Sciences, The University of Manchester, Manchester Academic Health Science Centre, Manchester, UK; Department of Rheumatology, Salford Royal Hospital, Northern Care Alliance NHS Foundation Trust, Salford, UK; Centre for Musculoskeletal Research, School of Biological Sciences, The University of Manchester, Manchester Academic Health Science Centre, Manchester, UK; Department of Rheumatology, Salford Royal Hospital, Northern Care Alliance NHS Foundation Trust, Salford, UK; Centre for Musculoskeletal Research, School of Biological Sciences, The University of Manchester, Manchester Academic Health Science Centre, Manchester, UK

Rheumatology key message

- Mobile phone thermography allows feasible, real-world collection of quantitative data about RP.

Dear Editor, Development of effective treatments for RP, including for SSc-related RP, has been hampered by the current lack of reliable outcome measures [1, 2]. Measuring outcome is challenging because RP ‘attacks’ generally occur out in the community (i.e. they cannot easily be observed in a hospital setting).

Mobile phone thermography (measuring skin surface temperature as an indirect measure of perfusion) could provide a way forward here, comparing favourably (including on test–retest reliability) with standard thermography [3] and with measurements strongly correlating with those obtained using a thermocouple (the gold standard, but impractical for community monitoring) [4]. We report a pilot study testing feasibility of mobile phone thermography as an ambulatory assessment tool. Specific objectives were to examine whether patients with RP could collect mobile phone thermography images out in the community, including during RP attacks, and whether it was possible to measure clinically relevant temperature changes from images taken during attacks.

Thirteen patients with SSc-related RP [all female, median age (range) 58 (32–71) years, median duration (range) of RP 12 (1–43) years, median (range) baseline Raynaud’s Condition Score (RCS) 1 (0–8)] [5] were recruited after signing informed consent. The study was approved by the North of Scotland Research Ethics Committee 2 (Reference: 21/NS/0042).

Patients attended twice at least 8 days apart. At the first visit, patients were given a FLIR One Pro thermal camera module (FLIR, Stockholm, Sweden) and compatible smartphone handset (Fig. 1a), and instructed on how to image their hands twice daily, morning and evening, when their hands were not affected by RP. For this feasibility study no specific instructions relating to hand-surface contact or to control of image backgrounds were given to patients. Patients were also asked to image RP attacks, with ideally at least three images (‘start, ‘middle’ and ‘end’) for each attack. They were also given a diary to record timing of daily images, details of any RP attacks and a daily RCS. At the second visit patients returned the handset and completed a feasibility questionnaire.

**Figure 1.**
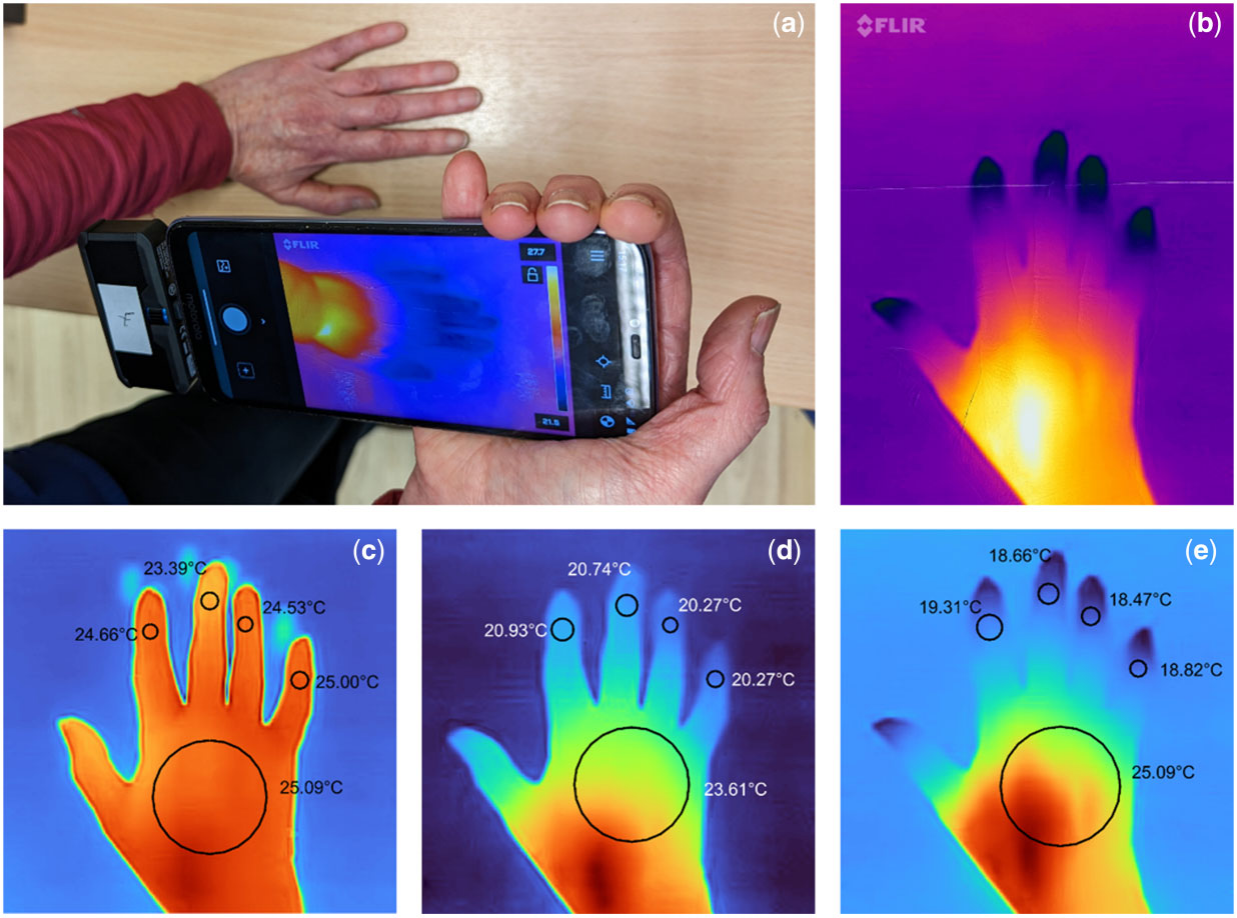
Imaging of RP attacks using the FLIR One smartphone-coupled thermal camera module. (**a**) The FLIR One thermal camera module used by patients in this study connected to a smartphone displaying real-time thermal images in the FLIR app; (**b**) a typical image from the FLIR One camera showing a hand during an RP attack as displayed in the FLIR app on the patient’s smartphone—darker/bluer areas are cold, lighter (red/yellow/white) areas are warmer; (c–e) a sequence of annotated images of a patient’s hand: the ‘normal’ hand (**c**) is followed ∼1 h later by initial stage of an RP attack (**d**) and then a few minutes later showing the peak of an RP attack (**e**). The images (c–e) were re-scaled and annotated using software in MATLAB which allowed the user to manually place regions of interest (ROIs) on each of the four distal fingers and on the dorsum of the hand as shown. For each ROI the temperatures were then extracted for further analysis

Suitable images (on the basis of whether the whole of the dorsum of the hand including digits could be seen) were manually annotated using custom software written in MATLAB [6] (see Fig. 1). Images were further classified as either ‘daily images’, or part of an RP attack, by matching up the image metadata with the patient-recorded information about RP attacks in the paper diaries.

Twelve patients (92%) completed the study (one lost to follow-up), returning 352 images in total (range 4–54), of which 251 were suitable for further analysis—176 were twice-daily images and 75 were RP attack images. Nine out of the 12 patients completing the study had recorded images during an RP attack.

Feasibility questionnaire responses (on a 10-point Likert scale; 1 very easy to 10 very difficult) were as follows [median (IQR)]: (i) ease of use of smartphone handset, 2 (1–4.5); (ii) ease of use of FLIR One thermal camera module, 3 (2–5.5); (iii) ease of use of FLIR app, 2 (2–6); (iv) difficulties due to the size of the task, 1 (1–2); (v) difficulties capturing images during an RP attack, 1 (1–7); and (vi) ease/difficulty of the study as a whole, 3 (1.5–5.5).

Mean (s.d.) non-attack temperatures (mornings and evenings combined) were 25.4 (0.2)°C for the digits and 28.4 (0.5)°C for the dorsum, and ‘during attack’ temperatures were: 22.3 (0.1)°C for the digits and 27.4 (0.9)°C for the dorsum. Including data from only those patients who imaged RP attacks (*n* = 9), the mean (s.d.) within-patient temperature difference (non-attack images minus RP attacks) for the digits was 3.9 (3.5)°C.

These results demonstrate that mobile phone thermography is a feasible method, including as rated by patients, for collecting rich, quantitative data about RP and importantly capturing RP episodes outside the clinic or hospital setting. The ability to detect differences in temperature due to RP attacks holds promise for developing new, sensitive outcome measures for clinical trials in RP. Further work on the sensitivity of this method to temperature changes induced by (for example) the introduction of vasodilator therapy is now required.

## Data Availability

The data underlying this article will be shared on reasonable request to the corresponding author.
